# Comparison of Grammar in Neurodevelopmental Disorders: The Case of Binding in Williams Syndrome and Autism With and Without Language Impairment

**DOI:** 10.1080/10489223.2013.766742

**Published:** 2013-02-06

**Authors:** Alexandra Perovic, Nadya Modyanova, Ken Wexler

**Affiliations:** University College London; Massachusetts Institute of Technology

## Abstract

This study investigates whether distinct neurodevelopmental disorders show distinct patterns of impairments in particular grammatical abilities and the relation of those grammatical patterns to general language delays and intellectual disabilities. We studied two disorders (autism and Williams syndrome [WS]) and two distinct properties (Principle A that governs reflexives and Principle B that, together with its associated pragmatic rule, governs pronouns) of the binding module of grammar. These properties are known to have markedly different courses of acquisition in typical development. We compare the knowledge of binding in children with autism with language impairment (ALI) and those with normal language (ALN) to that of children with WS, matched on age to the ALN group, and on age and nonverbal mental age (MA) to the ALI group, as well as to two groups of typically developing (TD) controls, matched on nonverbal MA to ALI and ALN groups. Our results reveal a remarkably different pattern of comprehension of personal pronouns and reflexives in ALI as opposed to ALN, WS, and two groups of TD controls. All five groups demonstrated an equal delay in their comprehension of personal pronouns, in line with widely reported delays in TD literature, argued to be due to delayed pragmatic abilities. However, and most strikingly, the ALI group also showed a pronounced difficulty in comprehension of reflexive pronouns, and particularly of the knowledge that the antecedent of a reflexive must c-command it. The revealed pattern confirms the existence of a particular impairment concerning Principle A in this module of grammar, unrelated to general language delays or cognitive deficits generally present in a large portion of individuals with autism as well as WS, or to general pragmatic deficits, known to be particularly prevalent in the population with autism.

## 1. INTRODUCTION

Binding is the linguistic submodule that guides our interpretation of reflexive and personal pronouns. Children make more co-reference errors on pronouns than on reflexives in the 3–6 years age range (for a review see Guasti 2002). The standard conclusion from experiments is that children know both Principle A (which regulates reflexives) and Principle B (which regulates pronouns), but that co-reference errors are made with pronouns due to a particular additional error: a pragmatic error for Chien & Wexler (1990), a processing/memory error for Grodzinsky & Reinhart (1993). The errors on Principle B are often called a *delay of Principle B effect (DPBE)*, a name that we will use for simplicity. More accurately, another name that has been used is *ADPBE*, where *A* stands for “apparent,” to reflect the fact that the error only *appears* to be a Principle B error, but is really something else. These questions of pragmatics/processing make it particularly compelling to study in populations known for pervasive pragmatic and suspected grammatical deficits, such as the disorders on the autism spectrum.

A recent investigation of binding in autism, reported in Perovic, Modyanova & Wexler (2012), revealed a striking pattern, rarely, if ever, observed in TD children in the literature. A small sample of children with autism (*n* = 14), aged 6–16 years, showed particular difficulties interpreting reflexive pronouns, unlike the two groups of control TD children matched for general levels of nonverbal abilities and grammar comprehension. Children with autism in this study demonstrated a better comprehension of pronouns than of the reflexives, but their comprehension of pronouns was still delayed, though comparable to both groups of TD matched controls. The revealed pattern indicates a particular deficit in the domain of grammar, previously not reported in autism spectrum disorders (ASD), and only noted so far in the population with Down syndrome (Ring & Clahsen 2005; Perovic 2006).

In view of particularly heterogeneous linguistic and cognitive abilities in the population with ASD, the simplest question to be asked is whether the deficient, or less developed, grammatical skills are unique to ASD, or whether they are the result of general language delay, intellectual disability, or impaired social skills associated with this disorder. For clinical populations generally, but in autism in particular, nonverbal IQ has been argued to be one of the most important prognostic variables (e.g., Szatmari, Bartolucci, Bremner, Bond & Rich 1989). On the other hand, recent research suggests that despite a general trend for the lower functioning individuals to be at a greater risk of delayed grammatical development, language impairment is to be found in a significant portion of individuals with ASD, regardless of the presence of intellectual impairment (e.g., Kjelgaard & Tager-Flusberg 2001). Cross-syndrome comparisons between populations that share some general characteristics such as language and developmental delays can be particularly useful in revealing the extent to which these factors affect the course of language acquisition in children with developmental disorders (Rice, Warren & Betz 2005). However, while early literature does report comparisons of the mastery of grammatical morphemes in spontaneous speech of children with ASD and Down syndrome, another population known for presence of both language delay and intellectual impairments (e.g., Tager-Flusberg et al. 1990), experimental studies directly comparing the knowledge of complex grammar in ASD to that of other developmental disorders are scarce.

To extend the findings of Perovic et al. (2012) further, and try to disentangle the role of different factors affecting grammatical development in ASD flagged in the preceding, here we investigate the knowledge of binding in a large sample of children with ASD, *n* = 48, and a sample of children with Williams syndrome (WS), an etiologically unrelated developmental disorder whose genetic basis is well understood and that shares the features of language delay and intellectual impairment frequently found in ASD, as well as deficits in social abilities (though these can be manifested in quite different ways; for more details see Klein-Tasman, Phillips, Lord, Mervis & Gallo 2009; Asada & Itakura 2012). This sample incorporates the 14 children from Perovic et al. (2012), though following the recent developments in the literature attempting to refine the language phenotypes of verbal children with autism (e.g., Kjelgaard & Tager-Flusberg 2001; Roberts, Rice & Flusberg 2004; Whitehouse, Barry & Bishop 2008), we divide the 48 children into two subgroups according to the presence or absence of language impairment: autism language impaired (ALI) and autism language normal (ALN). Their performance is compared to that of two TD control groups matched to each ASD group on gender and nonverbal mental age (MA), and a group of children with WS matched on chronological age (CA).

The results of the current study reveal a severe deficiency in interpreting reflexive pronouns, and a relatively better comprehension of personal pronouns in the ALI group, a pattern not observed in any of the TD control groups, the ALN or the WS group. The other groups all shared the pattern of an excellent performance on reflexives, and a relatively poorer performance on pronouns, in line with the so-called DPBE reported in typical development. The pattern of an equally good performance on pronouns in the children with ASD and WS, compared to MA-matched controls, suggests that general pragmatic difficulties pervasive in ASD are independent of their ability to interpret coreference. Crucially, the distinct patterns observed in the performance of the ALI versus WS group, who were matched in CA and nonverbal IQ, demonstrate that language delay and intellectual impairment, present in a large portion of individuals with either disorder, do not play a role in these children's ultimate linguistic achievement.

This article is organized as follows: In section 2.1 we present an overview of the knowledge of grammar in the disorders under investigation, and highlight the benefits of cross-syndrome comparisons in illuminating the perplexing heterogeneity of grammatical abilities in individuals with autism. In section 2.2 we introduce the module of binding and its acquisition. In section 3 we present our experimental investigation and its results, and in section 4 we discuss our findings and discuss the possible explanations of the patterns revealed in our clinical populations. Section 5 concludes the article.

## 2. BACKGROUND

### 2.1. Overview of Grammatical Development in WS and ASD

Developmental delays and intellectual impairment are present in a majority of individuals with WS, as well as a significant portion of children with ASD. In other respects, the two disorders seem to be completely the opposite of each other: children with WS are abnormally sociable while children on the autism spectrum are known to have difficulties in most basic social skills. Face processing skills are known to be a strength in WS, but not in ASD (for a review see Tager-Flusberg, Plesa Skwerer & Joseph 2006). Individuals with WS are known for a profound delay in visuospatial reasoning, while the opposite is the case in ASD (Mitchell & Ropar 2004).

However, when it comes to language, the picture is more complicated. Early studies pointed to WS as an archetypal example of a dissociation of language and cognition (e.g., by Bellugi and colleagues). Subsequent studies also reported findings suggesting an intact grammar in WS (Clahsen & Almazan 1998). More recent reports, however, carried out with a wider range of ages, control groups, and a detailed look at particular aspects of grammar, show that those aspects of grammar that mature early in typical development are relatively unimpaired (e.g., question formation: Zukowski 2001), but those that mature late in typical development (such as passives of psychological verbs and raising constructions) are exceptionally delayed, and possibly never acquired (Perovic & Wexler 2007; Perovic & Wexler 2010).

In autism, an impression that grammar is not particularly affected is widespread in the traditional literature (e.g., Lord & Paul 1997), though it is now becoming recognized that grammar may also be deficient in a sizeable proportion of these individuals. Grammatical morphology seems particularly compromised, especially finiteness marking (Kjelgaard & Tager-Flusberg 2001; Roberts et al. 2004), and the few experimental studies investigating complex syntactic structures report difficulties with passives (Tager-Flusberg 1981; Perovic, Modyanova & Wexler 2007), relative clauses (Riches, Loucas, Charman, Simonoff & Baird 2009), raising (Perovic et al. 2007) and subject control structures (Janke & Perovic submitted).

One of the perplexing topics in the literature is the heterogeneity of linguistic abilities in the population with autism. The deficits in communication, pragmatics and social aspects of language are present in *all* the individuals with autism, with this being the basis for the diagnosis of the disorder. However, the same is not the case with grammar. It has been argued that lower functioning individuals are more at risk of deficient grammar than higher functioning individuals, so much so that “language impairment and intellectual disability almost always co-occur together when associated with autism” (Boucher 2009:206). In children on the higher end of functioning on the spectrum, especially Asperger syndrome (AS), grammar has been reported to be mostly spared (Boucher 2009). Despite this general trend for the lower functioning individuals with autism to present with deficient grammar, the role of IQ in grammatical development in ASD is far from clear. Kjelgaard & Tager-Flusberg (2001) report that both intellectually impaired and unimpaired children with ASD scored low on a battery of standardized tests of grammar and vocabulary comprehension. Roberts et al. (2004) showed that regardless of their nonverbal IQ, a large proportion of autistic children in their study had difficulties with finiteness marking, the hallmark of the Optional Infinitive stage (Wexler 1994) and a suspected clinical marker for specific language impairment (SLI)—a population with no known intellectual deficits but with severe delays in the domain of grammar. The similarities in the language phenotypes between children with ASD and SLI, observed in both early and recent literature (for a review see Bishop 2003), are a matter of current debate: some studies argue for the comorbidity of autism and SLI (e.g., Lindgren, Folstein, Tomblin & Tager-Flusberg 2009), and others against (e.g., Whitehouse et al. 2008). One of the beneficial outcomes of this body of research is an effort to further refine the language phenotypes of a population known for heterogeneity of both linguistic and cognitive abilities. Following Tager-Flusberg and colleagues, it is now common in the literature to identify two major subgroups within the ASD population: children with autism whose performance on standardized language assessments is in the impaired range (autism + language impairment, thus ALI) and children with autism whose language seems normal on standardized measures (autism + normal language, thus ALN). Note, however, that this classification does not take into account the presence or absence of intellectual impairment. To assess the contribution of intellectual impairment to the ultimate linguistic achievement in clinical populations generally, cross-syndrome comparisons have proved particularly useful (Rice et al. 2005). However, with the exception of ASD versus SLI comparisons, we know of no experimental studies comparing complex grammar in ASD and other developmental disorders known for presence of intellectual impairments.

### 2.2. Binding and Its Acquisition

Binding refers to grammatical principles that regulate the distribution and interpretation of reflexive and personal pronouns. Binding Principle A, which constrains the domain of interpretation of reflexive pronouns, states that reflexive pronouns must be locally bound, that is, c-commanded and co-indexed, by their antecedent (Chomsky 1986).^1^ Thus, the referent of ‘herself’ in (1) can only be Sue, but not Mary:

(1) Mary_i_ says that Sue_j_ likes herself_j/*i_.

Principle B, which deals with the interpretation of personal pronouns (we won't deal with Principle C here), states that personal pronouns must not be locally bound, and thus the correct interpretation for ‘her’ in (2) is Mary, and not Sue:

(2) Mary_i_ says that Sue_j_ likes her_i/*j_.

Literature on typical development points to a robust pattern of a delayed comprehension of personal pronouns, up to the age of 6, in contrast to children's comprehension of reflexives, which is mastered at least by age 4—a phenomenon (misleadingly) termed the delay of Principle B effect (DPBE; for a review see, e.g., Guasti 2002; Elbourne 2005). The often-cited explanation (proposed by Chien & Wexler [1990] and endorsed by other researchers) invokes the different nature of constraints governing the interpretation of reflexive as opposed to personal pronouns. Reflexives are subject to syntactic binding, and always interpreted as bound variables. Since syntactic principles come online early, children generally have no difficulties applying syntactic principles and interpreting reflexive pronouns. Pronouns either can be interpreted as bound variables, when they are subject to syntactic binding, or they can have a co-referential interpretation, when they are subject to constraints that are extrasyntactic in nature.^2^ Chien & Wexler (1990) argue that these constraints are pragmatic and that children's errors are caused by particular difficulties with a pragmatic principle, called Principle P. Other explanations have been proposed: Grodzinsky & Reinhart (1993) accept Chien & Wexler's general account but argue for a particular rule of coreference and take children's difficulties to be some kind of memory or processing difficulty. Some recent studies point to difficulties in children's perspective taking (Hendriks & Spenader 2005/2006; which we take to be a particular version of Principle P), while a couple of studies argue that it was the pragmatic inadequacy of experimental materials used in the early literature that could be responsible for children's inability to comprehend pronouns correctly (Spenader, Smits & Hendriks 2009; Conroy, Lidz, Takahashi & Philips 2010). In our view, the Spenader et al. (2009) study suffers from an artifact of the particular stimulus used. An antecedent was not provided for both experimental possibilities. If children interpret a naming action as part of a single event described, as seems natural, then their results would be expected. Thus, the experiment fails to test Principle B; rather, it simply illustrates a natural pragmatic tendency. It is now clear that the Conroy et al. result is due to their wrong choice of pronoun in their stimuli (Hartman, Sudo & Wexler 2012). Inspection of the Conroy et al. audiotapes shows that they used a reduced, clitic form of the pronoun rather than the full pronoun. It is well known that clitics do not show a DPBE, and the theory of why is quite clear (Avrutin & Wexler 1992). Repeating their experiment using a clitic versus a full pronoun, Hartman et al. (2012) show in fact that the Conroy et al. material with a full pronoun elicits a strong DPBE, while the clitic form does not. Thus, these results are fully compatible with the classic view.

Regardless of differences in the methodology and approaches taken by more than 30 published studies investigating this phenomenon in the past two decades, there is a strong consensus that children's inability to interpret personal pronouns is a result of a delayed acquisition of extrasyntactic principles governing the co-referential interpretation of pronouns, which we have good reason to believe are pragmatic in nature.

What is known about binding in the syndromes we are interested in? Children with WS are reported to have no difficulties with reflexive binding (Ring & Clahsen 2005; Perovic & Wexler 2007). A slight delay of Principle B, comparable to TD controls matched for nonverbal and MA levels, was reported in the younger children with WS in Perovic & Wexler (2007), but not in teenagers with WS in Ring & Clahsen (2005).

The only study on binding in autism, our own investigation reported in Perovic et al. (2012), revealed a striking pattern, not observed in TD children matched for general levels of nonverbal abilities and grammar comprehension: a sample of 14 children on the lower end of functioning at the autism spectrum, aged 6–16, showed particular difficulties interpreting reflexive pronouns, but their interpretation of personal pronouns was in line with that of two groups of younger TD controls. Thus, in a picture selection task, upon hearing “Bart's dad is washing himself,” the children with autism chose the incorrect picture showing Bart's dad, Homer, washing Bart, about half of the time. Their comprehension of pronouns in sentences such as “Bart's dad is washing him” was *better* than on the reflexives and no different from that observed in either group of TD matched controls. This result is interesting for a variety of reasons. First, it reveals a deficit that is syntactic in nature—the explanation proposed by the authors is that children with autism do not show a sensitivity to c-command in establishing the complex syntactic dependency of binding, where the antecedent of a reflexive must c-command it. A deficit in the domain of complex syntactic dependencies supports recent research pointing to the presence of language impairment in a sizeable proportion of individuals with this disorder, contrary to the commonly accepted views that grammar is generally spared in autism. Second, in view of the widely recognized pragmatic deficits in the population on the autism spectrum, it is surprising that the sample of children in this study did not show a “stronger” DPBE, if it is the case that the constraints on coreference interpretation rely on some general pragmatic abilities.

While these results shed more light on our understanding of grammatical development in ASD, they also pose some very important questions. First, is the pattern of the deficient interpretation of the binding relation between the reflexive and its antecedent unique to autism, or is it a result of general language delay or intellectual disability also common in other developmental disorders? A related question is whether we should in fact expect a pattern of a *more* pronounced delay in the acquisition of constraints on coreference in children with ASD, known for generally impaired pragmatics, than in children with other etiologically unrelated developmental disorders of similar age, and thus a worse performance on pronouns in ASD than in children with a different developmental disorder. The third question deals with the huge heterogeneity of verbal and nonverbal skills within the autism spectrum—do all the children with ASD show the same pattern of deficient interpretation of reflexives but a better interpretation of pronouns, or can we distinguish between subgroups on the spectrum, and would the differences between these subgroups account for the differences in observed patterns?

The aim of the current study is to attempt to answer at least some of the important questions just posed, by investigating binding in a larger sample of children with ASD, both higher and lower functioning but grouped according to the presence or absence of language impairment as assessed by standardized measures of language abilities, following the now established classification in the literature. This would contribute to the literature on subgroups in ASD, currently an issue under much discussion, and would provide more insight into the debate as to whether language impairment exists or not in the ASD population. In order to control for factors such as the presence of language delay and intellectual impairment reported in a large portion of individuals with ASD, we include a sample of children with WS, another neurodevelopmental disorder known for general language and developmental delays.

## 3. THE CURRENT STUDY

### 3.1. Method

#### 3.1.1. Participants

One hundred and seventeen children participated in the study: 48 children with ASD (6 girls; CA: 6–18 years), 44 TD controls (8 girls, CA: 3;06–14;06), and 25 children with WS (13 girls, CA: 6–16). The details of their CA and scores on a battery of standardized measures of nonverbal and verbal abilities are given in Table 1: the Matrices subtest of Kaufman Brief Intelligence Test (KBIT), the Vocabulary subtest of KBIT (only for children with ASD), the Peabody Picture Vocabulary Test Third Edition (PPVT-3), and Test of Reception of Grammar Second Edition (TROG-2).

**TABLE 1:** Ages and Mean Scores (Standard Deviations) on Standardized Tests of Language and Cognition for the Five Participant Groups

*Group*	*ALI, n = 26*	*ALI TD, n = 24*	*ALN, n = 22*	*ALN TD, n = 22*	*WS, n = 25*	*Group Differences*
Mean age in months	142.43 (44.14)	75.23 (24.91)	134.33 (40.01)	121.95 (35.77)	139.32 (42.50)	TD ALI < TD ALN**, ALN***, ALL***, WS***
Range	77–218	44–128	73–222	54–201	72–200	
KBIT Matrices RS	16.79 (8.39)	17.90 (7.23)	30.09 (5.76)	29.77 (5.83)	19.76 (5.88)	ALI, TD ALI, WS < ALN***, TD ALN***
KBIT Matrices SS	66.92 (22.23)	101.50(9.41)	108.18 (15.45)	113.63(13.08)	74.24 (16.55)	ALI, WS < TD ALI***, ALN***, TD ALN***
KBIT Vocabulary SS	62.28 (20.47)		111.42(17.81)			ALI < ALN***
PPVT-III SS	58.5 (18.99)	108.0 (14.68)	111.86(18.07)	108.00(15.21)	82.08 (12.69)	ALI < WS*** < TD ALI***, ALN***, TD ALN***
TROG-2 SS	57.19 (4.73)	103.1 (17.31)	94.50 (12.25)	101.50(8.01)	74.29 (17.15)	ALI < WS*** < TD ALI***, ALN***, TD ALN***

*Note.* Measures on which relevant groups are matched: age in months for ALI, ALN and WS; KBIT Matrices raw score for ALI and ALI TD, and ALN and ALN TD, and KBIT Matrices raw and standard scores for ALI and WS. ALI = Autism Language Impaired; ALN = Autism Language Normal; TD = typically developing controls; WS = Williams syndrome; RS = raw score; SS = standard score.

*p < .05. **p < .01. ***p < .001.

Children with ASD, all with verbal communication skills, were selected from a larger pool of children in Modyanova, Perovic & Wexler (2011)^3^, and 14 of the 48 ASD children also took part in Perovic et al. (2012).^4^ They were recruited with the help of parent support groups in Massachusetts and Children's Hospital, Boston. All the children met the clinical *DSM–IV* criteria for the autism spectrum (APA 2000), and for 32 children who were also recruited for a study of defining phenotypic and genetic factors in ASD at Children's Hospital, Boston, as part of the Simons Simplex Collection (Lord et al. 2012), the diagnosis was confirmed by the Autism Diagnostic Interview–Revised (ADI-R; Lord et al. 2000) or Autism Diagnostic Observation Schedule (ADOS; Lord, Rutter & Le Couter 1994).^5^

In order to better control for the heterogeneity of abilities in our ASD sample that includes both low- and high-functioning children, following Tager-Flusberg and colleagues, we divided the children into two groups, autism language impaired (ALI, *n* = 26) and autism language normal (ALN, *n* = 22), according to their scores on the tests of receptive (PPVT-3, TROG-2) and productive language (Vocabulary subtest of KBIT) in our battery. To be categorized as language impaired, children's scores on at least two of the three language measures had to be below the 10th percentile (cf. Whitehouse et al. 2008).^6^

Table 1 shows the huge disparity in both verbal and nonverbal abilities in the two ASD groups, in line with other reports, for example, Roberts et al. (2004). The groups were by definition homogeneous (language-impaired vs. language normal) in terms of their scores on the standardized tests of language abilities: the scores of the ALI group are firmly in the impaired range on the three measures of grammar and vocabulary, whereas the opposite is true of the ALN group. With regard to their nonverbal IQ, children in the ALI group were generally on the lower end of functioning: KBIT Matrices SS *M* = 66.91 (*SD* = 22.22, range 40–105), but there was some heterogeneity in this group, with 5 of the 26 children having scores in the unimpaired range (KBIT Matrices SS: 97, 98, 100, 103 and 105). This is in contrast with the ALN group, whose mean KBIT Matrices SS was 108.18 (*SD* = 15.45, range 73–142), and with only one of the 22 children with an impaired nonverbal IQ: KBIT Matrices SS: 73. For comparison, note the scores of ALN versus ALI groups in Roberts et al. (2004) on nonverbal IQ: ALN group *M* = 95 (range 53–153), and ALI group *M* = 71.3 (range 43–102).

One control group consisted of children with WS, all with confirmed genetic diagnosis of WS, but excluding other diagnoses, such as ASD. They were recruited with the help of the Williams Syndrome Association from different states in the United States and their data was published in Perovic & Wexler (2007). The other two control groups consisted of TD children recruited from schools and day cares in Boston, MA, and the surrounding areas, with no signs of developmental delay reported by their teachers or parents, and with scores on standardized measures of nonverbal IQ, receptive vocabulary and receptive grammar all within the normal range.

TD control children were grouped into two relevant groups matched to the ALI and ALN groups on gender and on nonverbal reasoning (the raw scores of KBIT Matrices) and thus are referred to as TD ALI (*n* = 22)^7^ and TD ALN (*n* = 22).^8^ The ALI and ALN groups were matched to each other on CA and gender, and both were also matched to the WS group on CA. The ALI and the WS groups were also matched on nonverbal IQ (both the raw and standard scores of KBIT Matrices). All participants were native monolingual speakers of English and had no known concerns with their hearing.

#### 3.1.2. Experimental Materials and Procedure

The task used to test the comprehension of pronominal elements was a two-picture selection task, adapted from the original study of Wexler & Chien (1985), and also used in Perovic & Wexler (2007), and Perovic et al. (2012). Pictures were presented on the laptop computer and the child was asked to point to one of the two pictures which fit best with the sentence uttered by the experimenter. The pictures involved the characters from the Simpson family: Homer, Marge, Lisa, Bart and Maggie, recognizable to children of different ages. To make sure that the children are able to differentiate between the characters and understand the procedure of picture selection, the characters were first introduced to the child separately and in pairs, followed by pictures of the characters involved in one of the four actions described by the experimental verbs.

The verbs used were “wash,” “touch,” “point to” and “dress,” with each verb used twice. There were two experimental conditions, Name Pronoun (NPr; “Bart's dad is pointing to him”) Name Reflexive (NR; “Bart's dad is pointing to himself”), and two control conditions, Control Possessive (CP; “Bart's dad is petting a dog”) and Control Name (CN) (“Bart is pointing to Dad”). There were eight sentences per condition, totalling 32 sentences (see appendix for full list of sentences).

Possessive subjects were chosen in order to introduce two potential antecedents for the pronominal element: Bart's dad (= Homer), which c-commands the pronoun/reflexive, and Bart, the possessor, which does not c-command it. The CP condition was introduced in order to test the c-command part of the binding condition without any pronominal elements. Children were first tested on the battery of the standardized tests, followed by the experimental task. Probands were tested over one or two sessions, at their homes or schools, while TD controls were tested over two sessions at their schools or day care centers.

### 3.2. Results

The results were analyzed using the General Linear Mixed Model (GLMM), in SPSS 20 (for arguments that mixed regression models are more suitable to psycholinguistic research than the commonly used repeated measures analyses of variance see, e.g., Jaeger 2008; Quené & van der Bergh 2008). The fixed effects built into the model were Group (ALI, TD ALI, ALN, TD ALN, and WS), Sentence Type (NPr, NR, CP, and CN), and Group × Sentence Type interaction. The model revealed highly significant effects of Group, *F*(4, 448) = 27.63, *p* < .001, Sentence Type, *F*(3, 448) = 23.81, *p* < .001, and Sentence Type × Group interaction, *F*(12, 448) = 3.70, *p* < .001.

Estimated mean probabilities correct, for each sentence type and each group, are given in Table 2. Post hoc comparisons (Sidak corrected) allowed a further exploration of the performance on specific sentence types, both between groups and within each group.

**TABLE 2:** Estimated Mean Probabilities Correct (Standard Error) on the Target Items for Each Participant Group

	*ALI (SE)*	*ALI TD*	*ALN*	*ALN TD*	*WS*	*Intergroup Differences*
CN	0.79 (0.04)	0.98 (0.01)	0.98 (0.02)	0.98 (0.01)	0.94 (0.02)	ALI < TD ALI***, ALN***, TD ALN***, WS**
CP	0.77 (0.04)	0.96 (0.02)	0.99 (0.01)	0.98 (0.01)	0.93 (0.02)	ALI < TD ALI***, ALN***, TD ALN***, WS**
NPr	0.71 (0.05)	0.73 (0.05)	0.83 (0.04)	0.78 (0.05)	0.75 (0.05)	N.S.
NR	0.49 (0.05)	0.93 (0.03)	0.96 (0.02)	0.97 (0.02)	0.88 (0.03)	ALI < TD ALI***, ALN***, TD ALN***, WS***
Intragroup differences	NR < NPr**, CP***, CN***	NPr < NR**, CP***, CN***	NPr < NR*, CP**, CN**	NPr < NR**, CP***, CN***	NPr < NR |, CP**, CN**	

*Note.* CN = Control Name; CP = Control Possessive; NPr = Name Pronoun; NR = Name Reflexive; ALI = Autism Language Impaired; ALN = Autism Language Normal; TD = typically developing controls; WS = Williams syndrome.

| *p* < .10. *p < .05. **p < .01. ***p < .001.

A striking similarity was revealed in the performance of the four participant groups, ALN, WS and the two TD groups: no differences were observed between estimated mean probabilities correct on any of the sentence type, for any of these groups. The only between-group differences were revealed between the performance of the ALI group and each of the remaining four groups.

Estimated mean probabilities correct on the control condition CN for the ALI group (*M* = 0.79) were significantly worse compared to the ALN group (*M* = 0.98; *t*(448) = 4.261, *p* < .001), the TD ALI group (*M* = 0.98; *t*(448) = 4.465, *p* < .001), the TD ALN group (*M* = 0.98; *t*(448) = 4.465, *p* < .001) and the WS group (*M* = 0.94; *t*(448) = 3.303, *p* = .007).

On CP, the performance of the ALI group (*M* = 0.77) was worse than that of the ALN group (*M* = 0.99; *t*(448) = 5.579, *p* < .001), TD ALI group (*M* = 0.96; *t*(448) = 4.306, *p* < .001), TD ALN group (*M* = 0.98; *t*(448) = 5.118, *p* < .001), and the WS group (*M* = 0.93; *t*(448) = 3.594, *p* = .003).

On NPr, the performance of the ALI group (*M* = 0.71) did not differ significantly compared to ALN (*M* = 0.83), either of the TD groups, TD ALI (*M* = 0.73) and TD ALN (*M* = 0.78), or WS (*M* = 0.75).

The most striking difference between the ALI group and the remaining groups concerned their performance on the experimental condition NR. At *M* = 0.49, it was significantly worse than that of the ALN (*M* = 0.96; *t*(448) = 8.518, *p* < .001), TD ALI (*M* = 0.93; *t*(448) = 7.455, *p* < .001), TD ALN (*M* = 0.97; *t*(448) = 8.714, *p* < .001), and the WS group (*M* = 0.88; *t*(448) = 6.503, *p* < .001).

Furthermore, within-group comparisons revealed that the same experimental condition, NR, presented a particular difficulty to the ALI group compared to the other sentence types: their performance on NR was significantly worse than on each of the control conditions, CN (*t*(448) = 4.514, *p* < .001), CP (*t*(448) = 4.283, *p* < .001), as well as NPr (*t*(448) = 3.155, *p* = .008). In contrast, their performance on NPr did not differ significantly from either of the control conditions, CN or CP.

The opposite pattern was observed in the ALN and the remaining groups. If there was a difference between any of the conditions within each of the group, it concerned the worse performance on NPr, compared to the control conditions, and NR.

Thus, the ALN group performed worse on NPr than on CN (*t*(448) = 3.195, *p* = .007), CP (*t*(448) = 3.750, *p* = .001) and NR (*t*(448) = 2.709, *p* = .028). This pattern was repeated in the TD controls: the TD ALI group performed worse on NPr than on CN (*t*(448) = 4.809, *p* < .001), CP (*t*(448) = 4.236, *p* < .001), and NR (*t*(448) = 3.390, *p* = .003), as did the TD ALN group: NPr versus CN (*t*(448) = 4.024, *p* < .001), vsersus CP (*t*(448) = 4.046, *p* < .001), and versus NR (*t*(448) = 3.543, *p* = .002).

The WS group performed significantly worse on NPr than CN (*t*(448) = 3.654, *p* = .002), and CP (*t*(448) = 3.456, *p* = .003); however, the difference between NPr and NR only approached significance (*t*(448) = 2.290, *p* = .087).

In order to discount any concerns that the ALI children's poorer performance on NR is related to their inability to carry out the task, as reflected in their poorer performance on the control condition, CN, compared to the other four groups (a concern also raised in Perovic et al. 2012), following those authors we reran the analysis excluding all children performing less than 6/8 correct on this sentence type (for arguments that this cutoff is statistically appropriate see Perovic et al. 2012). After the exclusion of nine ALI children and one WS child, the estimated mean proportion correct on CN for the ALI group was 0.91. Crucially, the pattern remains the same: the GLMM analysis revealed significant main effects of Group *F*(4, 408) = 18.497, *p* < .001, Sentence Type *F*(3, 408) = 33.940, *p* < .001, and significant Group × Sentence Type *F*(12, 408) = 2.638, *p* = .002. Sidak corrected post hoc comparisons show that in this case, the ALI group's performance on CN is no longer different from that of the other three groups, but their performance on NR (*M* = 0.54) is still significantly worse than that of the four groups (all at *p* < .001). The lack of difference on NPr between groups remains, and the ALI group still performs worse on CP than any of the four groups (*p* < .001 compared to ALN and TD ALN, *p* = .003 compared to TD ALI, and *p* = .020 compared to WS).

Similarly, to check that the children's performance on NR was not related to their performance on the other control condition, CP, the analysis was also run excluding eight ALI children and one TD control with less than 6/8 correct on CP. Again, we see no difference in the patterns observed: the ALI group's estimated mean probability correct on NR (*M* = 0.52) was still significantly worse than that of the other groups (*p* < .001). The performance of the ALI group on CP (*M* = 0.92) was now only worse than the ALN group's (*p* = .015), probably due to ceiling effects (ALN: *M* = 0.99). The same difference on CP (*p* = .015) was observed between ALN and WS groups.

Examination of raw scores of individual children in the ALI group shows that 20 out of the 26 children in this group share the difficulties in comprehending NR sentences. Only six children reach 6, 7 or 8 out of 8 correct on NR, but 20 children score between 0 and 5 correct on this condition. This pattern contrasts starkly with the performance of children in the remaining groups: one out of 22 children in the ALN group scores 4 correct on NR, one TD control child out of 46 in both groups scores 5 correct, and 3 out of 25 WS children score 4 or 5 correct on NR.

## 4. DISCUSSION

In this section we first summarize the patterns observed in our five participant groups and discuss how they fit with the known picture of grammatical abilities in these populations. We also discuss the rather unexpected findings of an equal DPBE in all the groups, in view of our expectation, on a very rough model of pragmatic difficulties in ASD, of a more pronounced DPBE in ASD than in WS, as a result of known pragmatic deficits in the disorders on the spectrum. Recall, however, that children with WS also have pragmatic difficulties (e.g., Klein-Tasman et al. 2009), but it is generally accepted that difficulties in this domain are not the defining features of the syndrome, as they are in ASD. We will argue that the observed patterns suggest distinct linguistic profiles in ASD, and especially ALI, as opposed to WS, independent of any deficits in nonverbal abilities in these groups, as well as distinct profiles between ALI and ALN, independent from general deficits in pragmatics and in social cognition that all children with ASD share.

### 4.1. Distinct Patterns of Performance in ALI, ALN, WS, and TD Groups

The results of the TD children in our sample confirm the pattern widely reported in the literature: a significantly poorer performance on the NPr condition than on either of the control conditions CN and CP, as well as NR. Considering the chronological age of these two groups, with half of the children below the age of 6;6 (the approximate upper bound of the age at which DPBE is still observed), this was to be expected. These children show no difficulties with any of the other experimental conditions, thus showing a mastery of the syntactic constraint (c-command by the antecedent) governing reflexive binding.

As reported in Perovic & Wexler (2007), our WS group showed a pattern of a good performance on reflexives, consistent with reports of Ring & Clahsen (2005) on a TVJ task. This suggests that the knowledge that a reflexive must have a c-commanding antecedent is mastered early in WS, as it is in typical development. The only difficulty lay in the WS participants’ comprehension of pronouns (NPr) but this was not statistically significantly different from the performance observed in the two TD groups, the CA-matched ALN group and the CA- and MA-matched ALI group. The DPBE observed here was absent in the performance of WS participants studied in Ring & Clahsen (2005), which we attribute to these participants’ ages (all eight were teenagers).

Our ALI group showed two intriguing patterns. On NPr, their performance did not differ from that observed in each of the other experimental groups. However, the general pattern of performance on NPr was not the same as that observed in the other groups: all the other groups found NPr more difficult than the control conditions, CN and NP, as well as the reflexive condition, NR. The ALI group showed no difference between the NPr and the control conditions, CN and CP. The performance on NR is what crucially sets apart the ALI group from any of the other groups, however. First, it was the only group whose performance on NR was significantly worse than the NR performance in each of the other four groups; the other groups did not differ on this condition from each other. Second, the pattern the ALI group shows when comparing the performance on sentence types within the group itself was completely the reverse of that observed in the other groups: while the other groups performed better on NR than on NPr, the ALI group performed worse on NR than on NPr.

### 4.2. Knowledge of Principle A Is Compromised in ALI

The performance of the 26 participants with ALI in this study (14 of whom formed the sample studied in Perovic et al. [2012]) on NR signals a severe impairment in the interpretation of reflexives. On the basis of the expanded set of data with further children, it is reasonable to argue that Principle A is missing, or is incorrectly represented, in the grammar of the ALI population. Perovic at al. (2012) hypothesize that this difficulty is related to the c-command subpart of Principle A: that children with autism do not show a sensitivity to c-command in establishing the complex syntactic dependency of binding, where the antecedent of a reflexive must c-command the reflexive. It is not necessarily the case that children with ALI cannot compute c-command; they might be able to use it to constrain representations in other constructions. This would have to be tested in detail; it is probably too strong a failure to suggest.^9^ Rather, we will hypothesize that either children do not have Principle A or that they have some other version of it, for example, an ALI Principle A that says that the antecedent of a reflexive must be in the same clause as the reflexive. To test whether Principle A is completely missing or, for example, simply limits reflexives to having a clause-mate antecedent, we would have to test in the ALI population structures in which there are two antecedents, one local and one long-distant, as in the TD studies of Chien & Wexler (1990), experiment 2 or 3. To the best of our knowledge, this has not yet been done. For simplicity, let us hypothesize the least deficient possibility, namely, that the ALI version of Principle A constrains the ALI child only to having a clause-mate antecedent of the reflexive, missing the c-command part of Principle A.

Principle A is only restricted to anaphors. In view of the reports that knowledge of other complex syntactic dependencies also seems to be affected in autism (passives [Tager-Flusberg, 1981; Perovic et al. 2007] and raising [Perovic et al. 2007]), we may consider an alternative possibility, that the difficulties with the interpretation of reflexives, passives and raising in children with ASD be attributed to an imperfect knowledge of a constraint argued to underlie all three of these constructions, the A-chain Condition of Reinhart & Reuland (1993)^10^: “A maximal A-chain (a1 … an) contains exactly one link—a1—that is both [+R] and Case-marked” (696). The definition assumes that each link c-commands the next one in the chain. This A-chain Condition applies to all three constructions in question: all of them have a +R (referential) and Case-marked head, and a tail (a reflexive in the case of binding, an A-trace in the case of passives and raising) that is co-referential (intuitively “picks up” its reference from the head; in the case of binding) or vice versa (in the case of passives and raising). A missing, or perhaps incorrectly represented, A-chain Condition might cause the children in our experiment to violate the restriction that the head (antecedent) should c-command the tail (reflexive), and in fact allow the head to not c-command the tail.^11^ This may at first sight seem on the right track. However, for passives and raising the same does not hold. It is not as if children comprehend passives and raising structures, but don't know that a maximal A-chain contains one link that is [+R] and Case-marked. This deficit in the knowledge of the A-chain Condition would imply that children will understand (judge as grammatical) all sorts of ungrammatical passives and raising structures, say, super-raising, or nonlocal passives, for example, “*The book was expected John to buy,” alongside forms like “John was expected to buy the book.” They will have no problems, on the other hand, with simple passives and will understand them perfectly, for example. “John was kissed by Mary.” But to the best of our knowledge super-raising and nonlocal passives have never been observed in children, impaired or not.

In contrast, young TD children (e.g., Bever 1970; Maratsos, Fox, Becker & Chalkley 1985; Borer & Wexler 1987; Hirsch and Wexler 2006) and much older children with ASD (Tager-Flusberg 1981; Perovic et al. 2007) do not comprehend the verbal passive at all. In other words, both these predictions are false for children with ASD, TD children, and for any group that has been studied. The A-chain Condition is a filter, like Principle A: it rules out constructions. If the filter is missing or defective in a child, a too-broad range of structures will be accepted. So it is with reflexives in our study. But it is not the same with passives and raising: children fail to comprehend the passives or raised structures. The empirical results for reflexives, on the one hand, and passives and binding on the other (in ASD), do not allow the results to be put down to a deficiency of the A-chain Condition.

The data make it seem more likely that the ALI children do not have Principle A, which acts as a filter. They accept a too-wide class of structures. They know that a reflexive picks up its reference from its antecedent. However, they take a too-wide class of acceptable antecedents. The antecedent doesn't have to c-command the reflexive for such a child. Thus, this is a different type of problem than that raised by passives and raising. Principle A is missing in such children, not a far broader deficit.

It is in fact useful that the analysis shows that there has to be a difference in Principle A and A-chain type constructions such as passives and raising. This is because in TD children it has long been recognized that Principle A governing reflexives (especially the c-command part) is known by a very young age, whereas syntactic passives and raising take much longer to develop. This is the basis for the A-chain Development Hypothesis of Borer & Wexler (1987) and the Universal Phrase Requirement of Wexler (2004), which are taken to not apply to reflexives. So we must keep Principle A separate from the other constructions in order to explain TD data. But just on the basis of which constructions are delayed, we might now think that children with ALI show that all these constructions can be lumped together. The analysis shows that a more accurate look at the data (i.e., the *type* of nonknowledge) shows that this is not the case. Thus, it may turn out that not only the TD data but also the ALI data support the classic syntactic account, where Principle A and A-chain constructions cannot be reduced to exactly the same derivation. This should not be surprising. After all, reflexive binding constructions involve a co-referential relation between positions with distinct theta roles; the two DPs are different syntactic entities. In passives and raising, on the other hand, the co-referential relation involves two positions that share a theta role; in many respects they are the *same* syntactic entity. Our impression is that most approaches to syntactic theory still consider reflexive binding to be a very different operation from that of A-movement or even an LF kind of A-movement (i.e., a hidden movement). There are many syntactic differences between reflexive binding and A-chains. The child data show the joints rather explicitly.

What pattern of data in our experiment would we expect if children with ALI were missing Principle A, but instead had a principle that said that a reflexive must have an antecedent in the same clause, but there was no c-command condition relating the antecedent to the reflexive? Their use of a reflexive would now not be constrained; either antecedent would do. In our two-choice picture experiment, both pictures would be grammatical for the child. In, for example, “Bart's dad is pointing to himself,” the child missing Principle A would accept both “Bart” and “Bart's dad” as antecedents for “himself.” Faced with two pictures, both of which are grammatically interpretable, the child would have no basis for the choice. Lacking any kind of particular strategy, the simplest strategy would be a guess, yielding chance performance. The performance of our ALI group is remarkably close to chance: recall the already discussed individual data patterns, which show that most children with ALI performed at chance.

We might ask what would happen in a truth-value judgment task, in which the same sentence, “Bart's dad is pointing to himself,” is presented, along with one picture (the dad pointing to Bart in the picture, or the dad pointing to himself, the dad). Since both of these antecedents (Bart or Bart's dad) are grammatical for a child missing Principle A, the sentence can be interpreted as either true or false; it is ambiguous. In such situations, empirical data from many experiments show that there is a tendency for the child to say *yes* more often than *no*, that is, to more often than not find the reading that makes the sentence true. So in this case one might expect a greater than 50% yes response, although either response is possible and consistent with the child's grammar.

Why should it be Principle A that is missing in children with ASD? It is often thought that Principle A or, more generally, binding relations are part of the semantics, rather than part of the syntax. Perhaps this kind of semantic relation between entities is difficult for children with ALI. We do not have a more specific proposal as to why, but this might be a direction in which to look. One question to ask is: What do children with ALI know about bound variables? Do they understand them? Do they know that pronouns can be bound by quantifiers? Do they know that the quantifier that binds a variable must c-command it? Perhaps children with ASD have a general problem with bound variables, and this underlies why they don't seem to appreciate the c-command requirement for the antecedent of a reflexive. For now, these are only questions, but they do suggest empirical paths to follow in attempting to understand their striking difficulty with what is considered a basic property of reflexive pronouns.

One important question posed in Perovic et al. (2012) was whether the above deficit is present in the majority of individuals with autism. Our results here point to the negative answer. Only those individuals with autism who have been classified as having language impairment, ALI, on the basis of standardized measures of vocabulary and grammar, seem to show this deficit. The ALN group clearly demonstrates an unimpaired knowledge of this domain of grammar.

### 4.3. The Presence of DPBE in Atypical Development

Our findings suggest that all five groups comprehended sentences involving personal pronouns equally well—or, rather, equally poorly, considering that this was the only sentence type that proved difficult for the TD groups, as well as the ALN and WS groups.

At least for the TD population, we follow the typical literature in assuming that these difficulties are due to an inability to implement the constraints ruling out illicit coreference interpretation of pronouns, that is, the DPBE. It is not unreasonable to assume the same explanation for the atypically developing populations, especially those known for their language and cognitive delays, such as WS and ASD. On the other hand, it is unexpected to find an “equal” DPBE in all the experimental groups, considering the known pragmatic deficits in the disorders on the spectrum, which would suggest an even more pronounced DPBE in our sample of children with autism, ALI and ALN, than in WS, for example.

Do we have a good reason to believe that a DPBE is present equally in our groups? Let us consider the ALN group first. The estimated proportion correct for this group was the highest, at *M* = .83, after all. While the ALN children's performance was not different from that of the MA-matched control group, TD ALN, note that the two groups were also matched on CA, which makes the observed pattern perfectly typical; that is, the difficulties in the interpretation of pronouns in the ALN children was as expected, given their CA and general level of functioning. The ALI group, on the other hand, did not perform differently from their matched control group, TD ALI, but these children were only matched on nonverbal reasoning (the raw scores of KBIT Matrices), and not CA; thus, if the ALI group performs comparably well to much younger TD children, this certainly points to a “stronger” DPBE in the ALI group, at least. This argumentation may seem to be on the right track; however, no firm conclusions can be made. While the trend for the ALN to perform the highest on NPr seems obvious, recall that their performance was not significantly better from any of the groups included in the analysis: matched on nonverbal MA/CA or not, ALI, WS, or TD ALI.

In summary, what these results suggest is that whatever pragmatic abilities are involved in ruling illicit co-reference, they do not seem to be crucially impaired in any of the clinical samples in our study. Do these results shed any light on the nature of these pragmatic abilities? Schaeffer (2003) discusses differences between different kinds of pragmatics, a term that is used in many different ways. The pragmatics that relates to social rules may be differentially affected in children than the pragmatics that relates more directly to language, the pragmatics, for example, that is part of the governing conditions for reference. Our results thus show that the referential conditions that relate to the use of pronouns (e.g., Principle P of Chien & Wexler [1990]) are part of these “linguistic” conditions on pragmatics. The kind of conditions involved in the use of determiners may be another example of this kind of linguistic pragmatics, as a reviewer suggests. Could it be that linguistic pragmatics is not so affected in ASD? To our knowledge, not much has been studied with respect to the semantics and pragmatics of article use in ASD. Modyanova (2009) shows that correct use of determiners (with respect to semantics) is severely deficient in children with autism. Thus, we cannot say that linguistic pragmatics is not affected in autism, as long as the use of determiners involves linguistic pragmatics. Note, however, that Wexler (2010) argues that difficulties in TD children's use of determiners are in fact a semantic deficit, part of the (semantic part of) the grammar. If this is so, we see in the quite poor use of determiners in children with ASD a grammatical (semantic) deficit, and perhaps not a pragmatic deficit after all. To the extent this is true, the only case in which it can be argued that linguistic pragmatics has been directly studied in ASD is the Principle B/P case. So perhaps linguistic pragmatics is not affected in ASD, although syntax and semantics *are* affected. We do not wish to be firm in this conclusion. The data are limited so far on linguistic pragmatics in ASD. We put forth the suggestion as one possibility, to cover the data to date—clearly much more research is desirable.

### 4.4. Distinct Linguistic Profiles in ALI, ALN, and WS: What Is the Role of Language Delay and Intellectual Disability?

One of the questions we posed earlier concerns possible effects of a language delay on the knowledge of binding in children with ASD and WS in our study. We attempted to control for age-related effects by matching these clinical groups on age. While no individual language histories were obtained for our participants, we assume that their onset of language was generally delayed, as would be expected for these populations on the basis of the arguments presented in the literature.^12^

If language delay is present in WS, as well as ALI, it seems that it doesn't affect their knowledge of this aspect of grammar in the same way. The comprehension of pronouns seems delayed in both populations equally, but this was not the case for their performance on reflexives: the linguistic profiles in ALI and WS are markedly different.

The issue of intellectual disability is more complicated to unravel. What seems clear is that nonverbal IQ is not relevant when different groups are compared in our study. We attempted to control for the effect of nonverbal IQ by matching the ALI group to the TD children on the raw score of KBIT Matrices, while the ALI and the WS groups were matched on both raw and standard scores on this measure. Still the ALI group showed a different linguistic profile from either group, whereas the profile of the WS group was no different from that seen in TD (or ALN), despite substantially impaired intellectual abilities in WS compared to normal abilities in TD controls and ALN.

This leaves us with the issue of the role of nonverbal IQ within the ALI group. Can we say it is only low-functioning children with autism who show an atypical pattern? The general impression in the literature seems to be that lower functioning autistic children, that is, those with intellectual impairment, show a more delayed language than those who are high-functioning (Boucher 2009). It certainly is the case that the majority of the ALI children in our study also showed a clear intellectual impairment, despite some heterogeneity in this group. Crucially, the majority of children in the WS group were also intellectually impaired, but the pattern of a better performance on reflexives as opposed to pronouns in this group was as robust as in the TD controls. We thus believe that nonverbal IQ doesn't play much role in how children with ALI acquire grammar and, specifically, reflexive binding. These children, however, have been identified as being language impaired on several standardized measures of vocabulary and grammar, and their scores on these measures were significantly worse than those of the WS group. However, recall that reflexive binding is reported to be impaired in Down syndrome (Perovic 2006; Ring & Clahsen 2005). Like WS, this disorder is known for the presence of an extensive language delay and intellectual impairment, but compared to the relatively preserved aspects of grammar in WS, Down syndrome is also known for the presence of extensive syntactic impairments. Further work is needed in the field of cross-syndrome comparisons to shed more light on the similarities and differences in the linguistic profiles of children with distinct developmental disorders, and careful matching on different measures of verbal and nonverbal abilities is necessary. However, it seems clear that the ALI versus ALN classification of Tager-Flusberg and colleagues is supported outside the domain of standardized language measures, by our participants’ performance on an experimental task assessing complex grammar. More importantly, these findings confirm that grammatical impairment is present in a sizeable portion of the population with autism, with or without intellectual impairment. This result does not imply that ALI is autism plus SLI: children with SLI have been reported to show no difficulties with reflexive binding, only the ADPBE (van der Lely & Stollwerck 1997), suggesting that the linguistic characterizations of ALI and SLI seem to be quite different. We leave this issue for further research.

## 5. CONCLUSION

This study presents the first comparison of the knowledge of complex grammar in two subgroups of children with autism, those with and without general language impairment (ALI vs. ALN). The performance of the ALN group revealed an intact knowledge of reflexive binding, but a persisting pattern of difficulty with pronouns, a remnant of the so-called DBPE, despite the generally spared verbal and nonverbal abilities. The pattern of an atypical difficulty with reflexives in our larger sample of children with ALI, which incorporates the sample of children in Perovic et al. (2012), is even more striking here when compared to the CA-matched ALN group, and CA- and IQ-matched group with another neurodevelopmental disorder, WS.

By contrasting distinct neurodevelopmental disorders, we were able to probe further into the role of language delay, intellectual abilities and general pragmatic difficulties in the development of the linguistic submodule of binding. Language delay and poor intellectual abilities do not seem to play an important role in grammatical development in the populations investigated here; only the ALI group showed a deficit in reflexive binding, though both ASD and WS are known for general language and developmental delays. Social interaction difficulties and general pragmatic difficulties, reported in both ASD and WS, but more pervasive in ASD, also seem to play little role in these children's interpretation of coreference: the mastery of this aspect of linguistic knowledge in children with ASD and children with WS was no different from that of MA-matched TD controls.

## Footnotes

1. Node A in a phrase-marker c-commands node B if the lowest node that dominates A also dominates B.

2. That children are sensitive to the distinction between binding and co-reference is confirmed by their better performance on pronouns when bound by a quantified antecedent, which automatically excludes coreferential interpretation, as opposed to the referential antecedent (e.g., Chien & Wexler 1990). See Elbourne (2005) for the claim that some unpublished results (two sources) show that pronouns are difficult to interpret when bound by either referential or quantified antecedent. One of these publications, a dissertation, contains contradictory results with small sample sizes, and the other one we have not been able to obtain. The field standardly accepts the general result, in many replications.

3. Eleven more children with ASD were recruited but were excluded from the study: five children aged between 7 and 12 couldn't complete the task, five children younger than 6, and one 21 years old were excluded in order to keep the age range as close possible to that of the children in the other clinical group.

4. Note that all 14 participants recruited in Perovic et al. (2012) and included here are now classified as ALI; see below for details of reclassification of children with ASD according to general language abilities.

5. The diagnosis of AS was clinically confirmed for 15 of the children, with all but one functioning at the very high end of the spectrum. In view of the known difficulties between distinguishing high-functioning autism (HFA) and AS (Howlin 2003), the imminent elimination of the AS diagnosis from the *DSM–V* (for discussion see Worley & Matson 2012), and following the advice of two anonymous reviewers, we included both HFA and AS diagnoses in the current ALN sample.

6. There were seven children with scores in the impaired range only on one language measure, TROG-2. Four of these children (three with AS) had scores on the two vocabulary measures clearly in the average and even higher than average range: between 104 and 154, with TROG scores above 70: 74, 76, 76 and 78 (scores defined as “borderline” in Kjelgaard & Tager-Flusberg 2001). It was decided to classify these children as ALN. The remaining three children were classified as ALI, as their TROG SSs were particularly poor: 55 (below the 1st percentile), 67 (1st percentile), and 69 (in the 2nd percentile; scores defined as “impaired” in Kjelgaard & Tager-Flusberg 2001), and just about average on the other measures (SSs between 87 and 93, with one exception of a PPVT SS of 114).

7. The number of TD controls is lower than in the ALI group because we could not find matches for two ALI boys whose raw score on the KBIT Matrices was too low (0 and 3), and for two ALI boys who had not been administered the test.

8. As a result of their general functioning levels being in the unimpaired range on all available measures, the ALN group did not differ significantly to their controls on CA, KBIT SS, PPVT 3 raw scores, PPVT SS, and TROG 2 raw scores.

9. See the discussion soon to follow. We know of no evidence, for example, to suggest that TD children or children with ASD (or any other type of impairment) perform passive or raising movements to positions that don't c-command the trace of movement.

10. We are grateful to an anonymous reviewer for this suggestion.

11. We say *might* because it depends on how it is worked out that reflexives must be part of an A-chain. It really would not involve the A-chain Condition as stated, but rather the predictions that reflexives are part of a chain and that a chain involves c-commanding links. So perhaps an incorrectly represented A-chain Condition would mean that children don't know that the links in an A-chain c-command each other in order.

12. We do not make this argument for the ALN group, as this group consisted of children diagnosed with both HFA and AS, and the latter is argued not to involve language delays (Howlin 2003).

## APPENDIX 1

**Table:** Complete List of Sentences

1. Name Reflexive (NR) Bart's dad is touching himself.	Lisa's mom is touching herself. Bart's dad is pointing to himself. Lisa's mom is pointing to herself. Bart's dad is washing himself. Maggie's mom is washing herself. Maggie's mom is dressing herself. Lisa's mom is dressing herself.
2. Name Pronoun (NP) Bart's dad is touching him.	Lisa's mom is touching her. Bart's dad is pointing to him. Lisa's mom is pointing to her. Bart's dad is washing him. Maggie's mom is washing her. Maggie's mom is dressing her. Lisa's mom is dressing her.
3. Control Name (CN) Bart is pointing to Dad.	Lisa is touching Mom. Bart is washing Dad. Mom is dressing Maggie. Dad is pointing to Bart. Mom is touching Lisa. Mom is washing Maggie. Mom is dressing Lisa.
4. Control Possessive (CP) Lisa's mom is waving a flag.	Bart's dad is petting a dog. Maggie's mom is petting a dog. Lisa's mom is driving a car. Lisa's mom is playing with blocks. Bart's dad is eating an ice cream. Maggie's mom is eating an ice cream. Bart's dad is licking a lamp post.

## References

[R1] American Psychiatric Association (2000). The diagnostic and statistical manual of mental disorders.

[R2] Asada Kosuke, Itakura Shoji (2012). Social phenotypes of autism spectrum disorders and Williams syndrome: Similarities and differences. Frontiers in Psychology.

[R3] Avrutin Sergey, Wexler Ken (1992). Development of Principle B in Russian: Coindexation at LF and coreference. Language Acquisition.

[R4] Bellugi Ursula, Marks Shelly, Bihrle Amy, Sabo Helene, Bishop Dorothy, Mogford Kay (1988). Dissociation between language and cognitive functions in Williams syndrome. Language development in exceptional circumstances.

[R5] Bever Tom, Hayes J. R. (1970). The cognitive basis for linguistic structure. Cognitive development of language.

[R6] Bishop Dorothy, Bock Gregory, Goode Jamie (2003). Autism and specific language impairment: categorical distinction or continuum?. Autism: neural basis and treatment possibilities (Novartis Foundation Symposium 251).

[R7] Borer Hagit, Wexler Ken, Roeper T., Williams E. (1987). The maturation of syntax. Parameter setting.

[R8] Boucher Jill (2009). The autistic spectrum: Characteristics, causes and practical issues.

[R9] Chien Yu-Chin, Wexler Ken (1990). Children's knowledge of locality conditions in binding as evidence for the modularity of syntax and pragmatics. Language Acquisition.

[R10] Chomsky Noam (1986). Knowledge of language: Its nature, origin and use.

[R11] Clahsen Harald, Almazan Mayella (1998). Syntax and morphology in Williams syndrome. Cognition.

[R12] Conroy Anastasia, Takahashi Eri, Lidz Jeffrey, Phillips Colin (2010). Equal treatment for all antecedents: How children succeed with Principle B. Linguistic Inquiry.

[R13] Elbourne Paul (2005). On the acquisition of Principle B. Linguistic Inquiry.

[R14] Grodzinsky Yosef, Reinhart Tania (1993). The innateness of binding and coreference. Linguistic Inquiry.

[R15] Guasti Maria Teresa (2002). Language development: The growth of grammar.

[R16] Hartman Jeremy, Sudo Yasutada, Wexler Ken (2012). Principle B and phonologically reduced pronouns in child English. Paper presented at Generative Approaches to Language Acquisition-North America (GALANA) 5.

[R17] Hendriks Petra, Spenader Jennifer (2005/2006). When production precedes comprehension: An optimization approach to the acquisition of pronouns. Language Acquisition.

[R18] Hirsch Christopher, Wexler Ken, Ud Deen Kamil, Nomura Jun, Schulz Barbara, Schwartz Bonnie D. (2006). Children's passives and their resulting interpretation. The Proceedings of the Inaugural Conference on Generative Approaches to Language Acquisition-North America.

[R19] Howlin Patricia (2003). Outcome in high-functioning adults with autism and without early language delays: Implications for the differentiation between autism and Asperger syndrome. Journal of Autism and Developmental Disorders.

[R20] Jaeger T. Florian (2008). Categorical data analysis: Away from ANOVAs (transformation or not) and towards Logit Mixed Models. Journal of Memory and Language.

[R21] Janke Vikki, Perovic Alexandra Interpretation of infinitives: a first insight from high functioning autistic (HFA) individuals. International Journal of Language & Communication Disorders.

[R22] Kjelgaard Margaret, Tager-Flusberg Helen (2001). An investigation of language impairment in autism: Implications for genetic subgroups. Language and Cognitive Processes.

[R23] Klein-Tasman Bonita, Phillips Kristin D., Lord Catherine, Mervis Carolyn B., Gallo Frank J. (2009). Overlap with the autism spectrum in young children with Williams syndrome. Journal of Developmental & Behavioral Pediatrics.

[R24] Lindgren Kristen, Folstein Susan, Bruce Tomblin, Tager-Flusberg Helen (2009). Language and reading abilities of children with autism spectrum disorders and their first degree relatives. Autism Research.

[R25] Lord Catherine, Paul Rhea, Cohen Donald J., Volkmar Fred R. (1997). Language and communication in autism. Handbook of autism and pervasive development disorders.

[R26] Lord Catherine, Risi Susan, Lambrecht Linda, Cook Edwin H., Leventhal Bennett L., DiLavore Pamela C., Pickles Andrew, Rutter Michael (2000). The Autism Diagnostic Observation Schedule-Generic: A standard measure of social and communication deficits associated with the spectrum of autism. Journal of Autism and Developmental Disorders.

[R27] Lord Catherine, Rutter Michael, Le Couter Anne (1994). Autism Diagnostic Interview-Revised: A revised version of a diagnostic interview for caregivers of individuals with possible pervasive developmental disorders. Journal of Autism and Developmental Disorders.

[R28] Lord Catherine, Petkova Eva, Hus Vanessa, Gan Weijin, Lu Feihan, Martin Donna M., Ousley Opal, Guy Lisa, Bernier Raphael, Gerdts Jennifer (2012). A multisite study of the clinical diagnosis of different autism spectrum disorders. Archives of General Psychiatry.

[R29] Maratsos Michael, Fox Dana E. C., Becker Judith A., Anne Chalkley Mary (1985). Semantic restrictions on children's passives. Cognition.

[R30] Mitchell Peter, Ropar Danielle (2004). Visuo-spatial abilities in autism: A review. Infant and Child Development.

[R31] Modyanova Nadezhda (2009). Semantic and pragmatic language development in typical acquisition, autism spectrum disorders, and Williams syndrome with reference to developmental neurogenetics of the latter.

[R32] Modyanova Nadezhda, Perovic Alexandra, Wexler Ken (2011). Binding in high- and low-functioning children with autism spectrum disorders.

[R33] Perovic Alexandra (2006). Syntactic deficit in Down syndrome: More evidence for the modular organization of language. Lingua.

[R34] Perovic Alexandra, Wexler Ken (2007). Complex grammar in Williams syndrome. Clinical Linguistics and Phonetics.

[R35] Perovic Alexandra, Modyanova Nadezhda, Wexler Ken (2007). Knowledge of c-command and A-movement in children and adolescents with autism and with Asperger syndrome.

[R36] Perovic Alexandra, Wexler Ken (2010). Development of verbal passive in Williams syndrome. Journal of Speech, Language and Hearing Research.

[R37] Perovic Alexandra, Modyanova Nadezhda, Wexler Ken (2012). Comparison of reflexive and personal pronouns in children with autism: A syntactic or pragmatic deficit?. Applied Psycholinguistics.

[R38] Quené Hugo, van der Bergh Huub (2008). Examples of mixed effects modeling with crossed random effects and with binomial data. Journal of Memory and Language.

[R39] Reinhart Tanya, Reuland Eric (1993). Reflexivity. Linguistic Inquiry.

[R40] Rice Mabel L, Warren Steven F., Betz Stacy K. (2005). Language symptoms of developmental language disorders: An overview of autism, Down syndrome, fragile X, specific language impairment, and Williams syndrome. Applied Psycholinguistics.

[R41] Riches Nick, Loucas Tom, Charman Tony, Simonoff Emily, Baird Gillian (2009). Sentence repetition in adolescents with specific language impairments and autism: An investigation of complex syntax. International Journal of Language and Communication Disorders.

[R42] Roberts Joseph, Rice Mabel, Tager-Flusberg Helen (2004). Tense marking in children with autism. Applied Psycholinguistics.

[R43] Ring Melanie, Clahsen Harald (2005). Distinct patterns of language impairment in Down's syndrome, Williams syndrome, and SLI: The case of syntactic chains. Journal of Neurolinguistics.

[R44] Schaeffer Jeannette, Levy Yonata, Schaeffer Jeannette (2003). Pragmatics and SLI. Language competence across populations: Towards a definition of specific language impairment.

[R45] Spenader Jennifer, Smits Erik-Jan, Hendriks Petra (2009). Coherent discourse solves the pronoun interpretation problem. Journal of Child Language.

[R46] Szatmari Peter, Bartolucci Giampiero, Bremner Rebecca, Bond S., Rich S. (1989). A follow up study of high-functioning autistic children. Journal of Autism and Developmental Disorders.

[R47] Tager-Flusberg Helen (1981). On the nature of linguistic functioning in early infantile autism. Journal of Autism and Developmental Disorders.

[R48] Tager-Flusberg Helen, Calkins Susan, Nolin Tina, Baumberger Therese, Anderson Marcia, Chadwick-Dias Ann (1990). A longitudinal study of language acquisition in autistic and Down syndrome children. Journal of Autism and Developmental Disorders.

[R49] Tager-Flusberg Helen, Plesa-Skwerer Daniela, Joseph Robert (2006). Model syndromes for investigating social cognitive and affective neuroscience: A comparison of autism and Williams syndrome. Social Cognitive and Affective Neuroscience.

[R50] van der Lely Heather K. J., Stollwerck Linda (1997). Binding theory and specifically language impaired children. Cognition.

[R51] Wexler Ken, Lightfoot David, Hornstein Norbert (1994). Optional infinitives, head movement, and the economy of derivations. Verb movement.

[R52] Wexler Ken (2004). Theory of phasal development: Perfection in child grammar. MIT Working Papers in Linguistics.

[R53] Wexler Ken, Gibson Edward, Pearlmutter Neal J. (2010). Cues don't explain learning: Maximal trouble in the determiner system. The processing and acquisition of reference.

[R54] Wexler Ken, Chien Yu-Chin (1985). The development of lexical anaphors and pronouns. Papers and Reports on Child Language Development.

[R55] Whitehouse Andrew, Barry Johanna, Bishop Dorothy (2008). Further defining the language impairment of autism: Is there a specific language impairment subtype?. Journal of Communication Disorders.

[R56] Worley A. Julie, Matson Johnny L. (2012). Comparing symptoms of autism spectrum disorders using the current DSM-IV-TR diagnostic criteria and the proposed DSM-V diagnostic criteria. Research in Autism Spectrum Disorders.

[R57] Zukowski A. (2001). Uncovering grammatical competence in children with Williams syndrome.

